# *KRAS*-driven model of Gorham-Stout disease effectively treated with trametinib

**DOI:** 10.1172/jci.insight.149831

**Published:** 2021-08-09

**Authors:** Nassim Homayun-Sepehr, Anna L. McCarter, Raphaël Helaers, Christine Galant, Laurence M. Boon, Pascal Brouillard, Miikka Vikkula, Michael T. Dellinger

**Affiliations:** 1 Human Molecular Genetics, de Duve Institute, University of Louvain, Brussels, Belgium.; 2 Division of Surgical Oncology, Department of Surgery and Hamon Center for Therapeutic Oncology Research, UT Southwestern Medical Center, Dallas, Texas, USA.; 3 Center for Vascular Anomalies, Division of Pathology, and; 4 Division of Plastic Surgery, Cliniques universitaires Saint-Luc, University of Louvain, European Reference Network for Rare Multisystemic Vascular Diseases, Vascular Anomalies Working Group, European Reference Centre, Brussels, Belgium.; 5 Walloon Excellence in Life Sciences and Biotechnology, University of Louvain, Brussels, Belgium.; 6 Department of Molecular Biology and Hamon Center for Regenerative Science and Medicine, UT Southwestern Medical Center, Dallas, Texas, USA.

**Keywords:** Angiogenesis, Vascular Biology, Bone disease, Molecular genetics

## Abstract

Gorham-Stout disease (GSD) is a sporadically occurring lymphatic disorder. Patients with GSD develop ectopic lymphatics in bone, gradually lose bone, and can have life-threatening complications, such as chylothorax. The etiology of GSD is poorly understood, and current treatments for this disease are inadequate for most patients. To explore the pathogenesis of GSD, we performed targeted high-throughput sequencing with samples from a patient with GSD and identified an activating somatic mutation in *KRAS* (p.G12V). To characterize the effect of hyperactive KRAS signaling on lymphatic development, we expressed an active form of *KRAS* (p.G12D) in murine lymphatics (*iLEC^Kras^* mice). We found that *iLEC^Kras^* mice developed lymphatics in bone, which is a hallmark of GSD. We also found that lymphatic valve development and maintenance was altered in *iLEC^Kras^* mice. Because most *iLEC^Kras^* mice developed chylothorax and died before they had significant bone disease, we analyzed the effect of trametinib (an FDA-approved MEK1/2 inhibitor) on lymphatic valve regression in *iLEC^Kras^* mice. Notably, we found that trametinib suppressed this phenotype in *iLEC^Kras^* mice. Together, our results demonstrate that somatic activating mutations in *KRAS* can be associated with GSD and reveal that hyperactive KRAS signaling stimulates the formation of lymphatics in bone and impairs the development of lymphatic valves. These findings provide insight into the pathogenesis of GSD and suggest that trametinib could be an effective treatment for GSD.

## Introduction

Gorham-Stout disease (GSD) is a sporadically occurring disorder of the lymphatic system (1). Patients with GSD have retrograde lymph flow, irregular lymphatic vessels in their soft tissues, and osteolytic lesions that contain lymphatic vessels (1). In severe cases of GSD, the osteolytic process continues until entire bones are lost and replaced by fibrous tissue (2). Approximately 300 cases of GSD have been described since Gorham and Stout published their landmark paper in 1955 (2, 3). These reports have revealed that GSD is typically diagnosed in children and young adults and that it does not display any clear sex or inheritance pattern (3). Although GSD can affect any bone in the body, it most frequently affects the ribs and vertebrae (3). Unfortunately, patients with thoracic involvement tend to develop chylothorax, a complication that can cause respiratory distress, failure, and death (4). Depending on the location and severity of the disease, several different strategies are used to treat GSD. The most common treatments include surgery, sclerotherapy, bisphosphonates, and sirolimus (3, 5). However, these treatments are not curative and can be inadequate for patients (6, 7). Therefore, there is an urgent need for new treatments for GSD.

A better understanding of the etiology of GSD could lead to new treatments for patients. Lymphatic and vascular anomalies are caused by errors in the development of the lymphatic and vascular systems. Since the first demonstration in 2009 that sporadically occurring venous malformations are caused by somatic activating TIE2 mutations, various sporadically occurring lymphatic and vascular anomalies have been associated with somatic activating mutations (8). The majority of these mutations are identical to those found in oncogenes in cancers. For example, isolated lymphatic malformations and some generalized lymphatic anomalies have been associated with somatic activating *PIK3CA* mutations (9). The cause of GSD, however, has remained unknown.

Here, we report the identification of a somatic activating hotspot mutation in *KRAS* (p.G12V) in tissues from a patient with GSD. We also show that hyperactive KRAS signaling in lymphatic endothelial cells (LECs) in mice stimulates the development of lymphatic vessels in bone, impairs the development of lymphatic valves, and causes chylothorax. Additionally, we show that an FDA-approved MEK1/2 inhibitor, trametinib, suppresses lymphatic valve regression in our mouse model. Together, these findings pinpoint RAS signaling in the etiology of GSD and suggest that MEK inhibitors could be used as a treatment for this life-threatening disease.

## Results

### Clinical description of patient with GSD.

**Figure 1:**
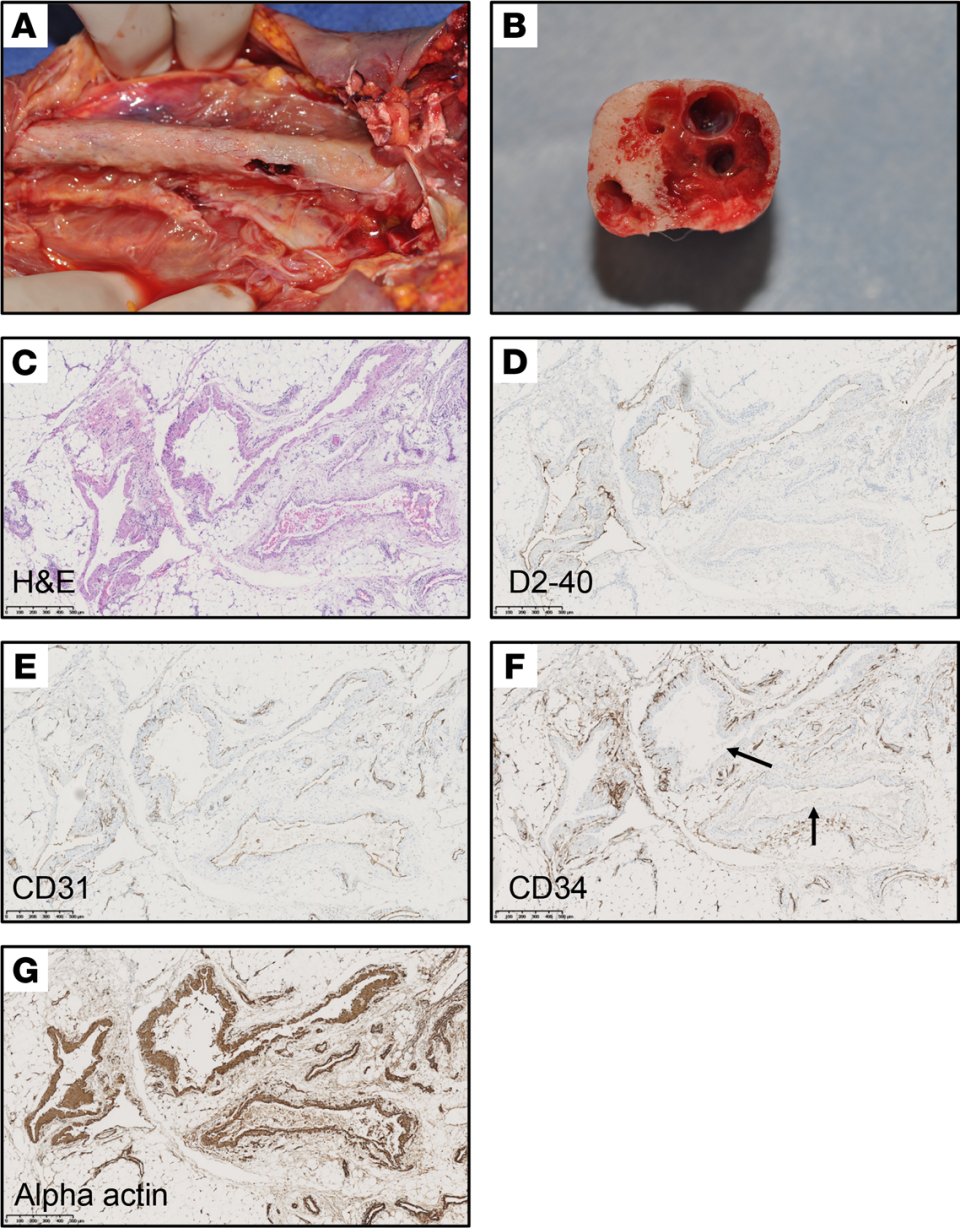
Patient and histology. (**A**) Global view and (**B**) transverse section of the humerus invaded and destroyed by lymphatic vessels. (**C–G**) Histology and immunohistochemistry. (**C**) Histological examination with hematoxylin and eosin staining demonstrated an increase in the size and number of irregular thin-walled channels, made mainly of interconnecting and dilated lymphatic spaces. (**D** and **E**) The single internal layer of these vessels was positive for D2-40 (antibody that detects podoplanin) and for CD31, also known as PECAM1, demonstrating their lymphatic origin. (**F**) Immunostaining for CD34. The arrows point to CD34-negative lymphatics. (**G**) Smooth muscle α actin highlighted the irregular muscular walls. Scale bar: 500 μm (**C–G**).

The index patient received a diagnosis of GSD after she presented with osteolytic lesions and intraosseous lymphatic malformations. Lytic lesions were seen on her right clavicle, humerus, radius, and cubitus as well as her right femur, tibia, and the D10 vertebra. Her parents had no symptoms. She had pathologic humeral fractures at the ages of 6 and 8 years. In adulthood, she suffered from chronic ascites with intestinal lymphangiectasias causing exudative enteropathy by the age of 33 years. She experienced progressive necrosis of the right wrist and the forearm, which led to transhumeral amputation at the age of 36 years. Macroscopically, the medullary part of the resected humerus was vacuolated (Figure 1, A and B). Microscopically, tissue between bone trabeculae showed irregular interconnecting and dilated lymphatic and vascular channels. Most of the vessels were lined by a single layer of endothelial cells positive for D2-40 (antibody that recognizes podoplanin) and CD31 and negative for CD34, demonstrating their lymphatic origin (Figure 1, C–G). The patient had been treated with several medications, including IFN alfa2a, thalidomide, sunitinib, and, finally, with sirolimus, all with minor or no response. She passed away because of fulminant sepsis at the age of 37 years.

### Identification of a KRAS oncogenic mutation in GSD.

**Figure 2:**
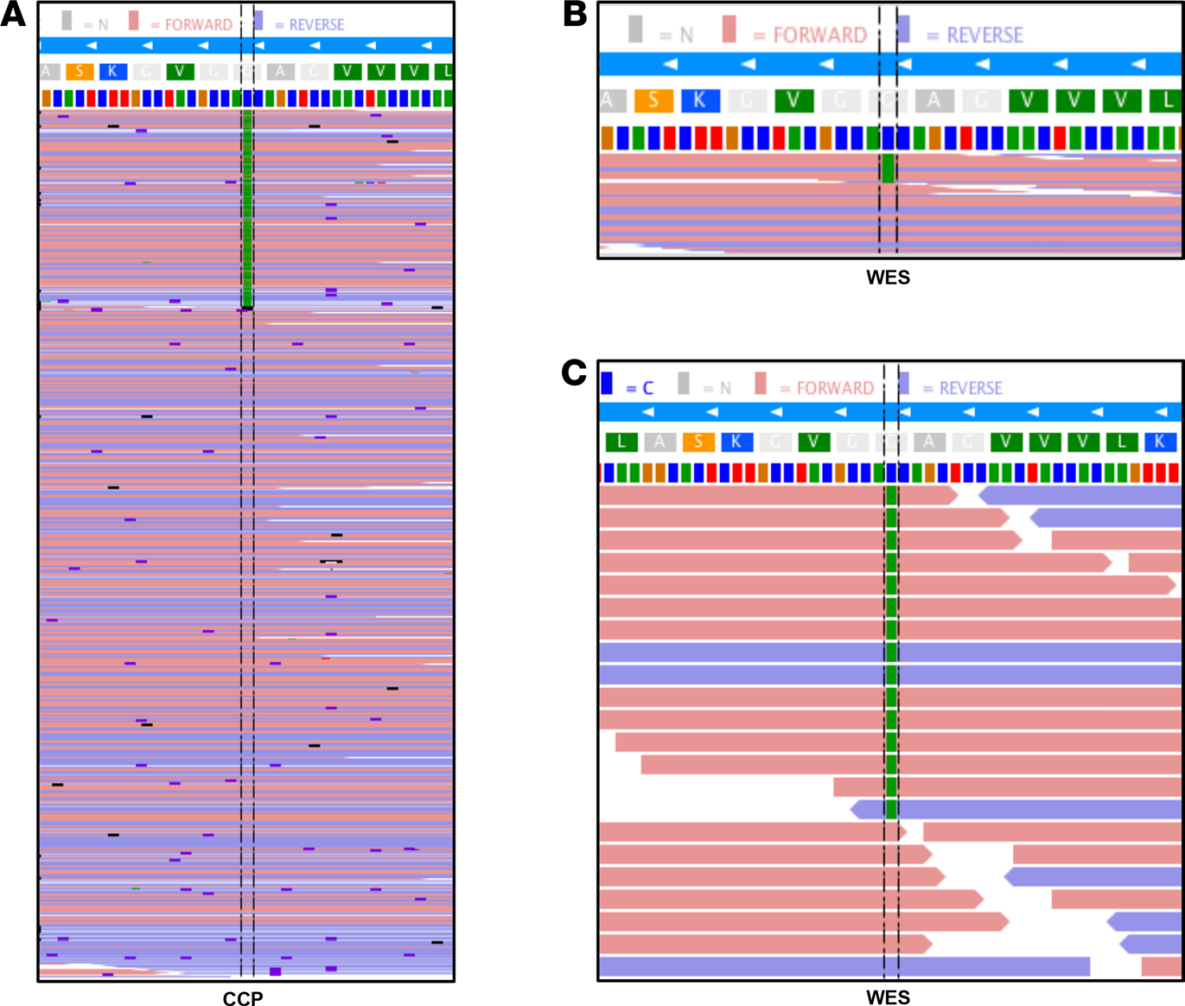
*KRAS* oncogenic mutation in 1 patient with GSD. (**A** and **B**) The *c.35G>T*; p.G12V somatic mutation in *KRAS* is present in forward and reverse reads (red and blue, respectively) and (**A**) represents 23% of the alleles in the comprehensive cancer panel (142 of 620 reads) and (**B**) 28% of the alleles in whole-exome sequencing (WES; 15 of 55 reads). Nucleotides are represented by colors (T, red; G, brown; A, green; C, blue). (**C**) Zoomed view of WES data.

Our hypothesis was that an oncogenic mutation should be the cause of GSD, as seen in other vascular malformations. We first used a comprehensive cancer panel to sequence 408 cancer-related genes at high median coverage (>2000×). We discovered 1 heterozygous nucleotide substitution (*c.35G>T*, p.G12V) in *KRAS* in tissue resected from a bone invested with abnormal lymphatics. This mutation was present in 23% of the alleles (142 reads of 620 reads) (Figure 2A). The G12V is a well-known oncogenic mutation in *KRAS* (reported 10,797 times in COSMIC, the database of somatic mutations in cancers).

To exclude changes in other genes, we subsequently performed whole-exome sequencing (WES) on tissue and blood DNA of the patient (with average coverage of 200× for tissue and 70× for blood). Paired analysis using Mutect2 (10) confirmed the *KRAS* mutation in 28% of the reads (15 reads of 55) in the tissue (Figure 2, B and C). No additional somatic or germline mutation that we could consider as being potentially disease causing was detected, including the genes known to be mutated in various lymphatic and vascular malformations*,* such as *PIK3CA* or *NRAS*. FACETS (11) and ExomeDepth (12) software programs did not reveal any copy number alterations in the WES data.

### iLEC^Kras^ mice develop ectopic lymphatics in bone.

**Figure 3:**
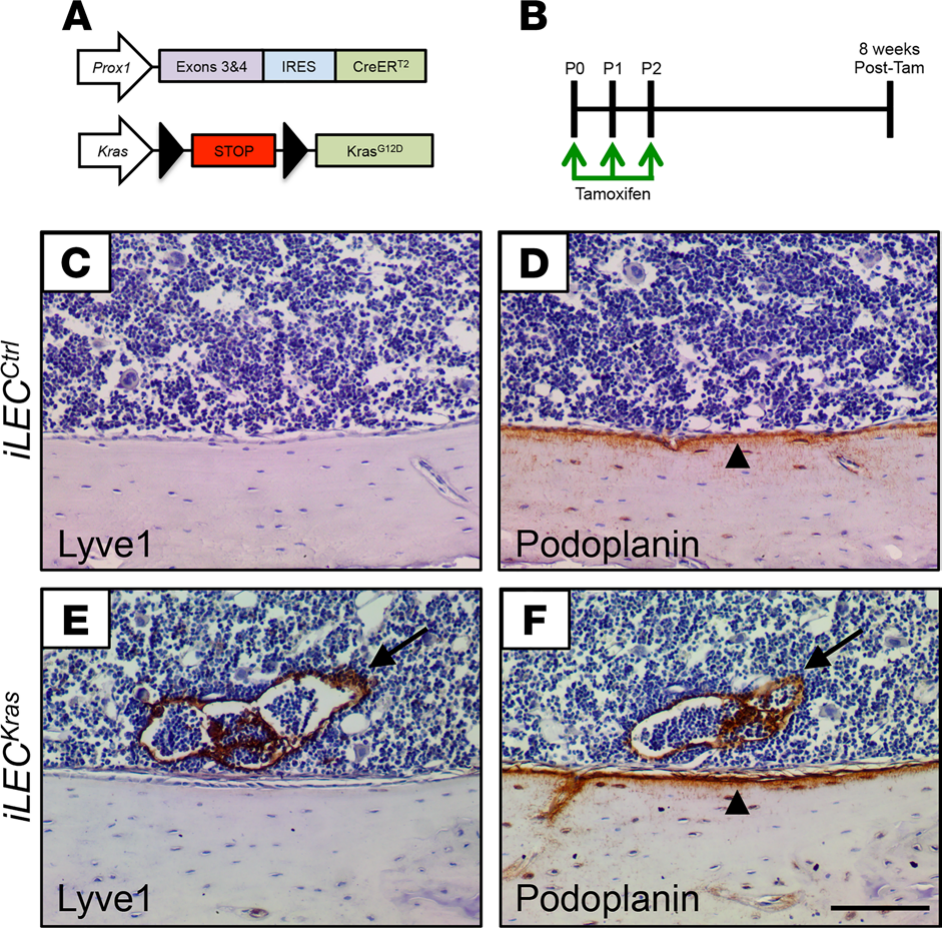
*iLEC^Kras^* mice develop ectopic lymphatics in bone. (**A**) Schematics of the *Prox1-CreER^T2^* and *Kras^LSL-G12D^* alleles. (**B**) Schematic showing when mice were fed tamoxifen (2 μl of 25 mg/ml solution). Femurs and tibias were collected when mice were 8 weeks old. (**C–F**) Bones from *iLEC^Ctrl^* mice (*n* = 10; **C** and **D**) and *iLEC^Kras^* mice (*n* = 3; **E** and **F**) were stained with antibodies against Lyve1 and podoplanin. Bones from *iLEC^Ctrl^* mice did not contain lymphatics. In contrast, bones from 2 of 3 *iLEC^Kras^* mice contained lymphatics (arrows). Bones from *iLEC^Ctrl^* and *iLEC^Kras^* contained numerous podoplanin-positive osteocytes (arrowheads). Scale bar: 100 μm.

Because GSD is a lymphatic anomaly, we set out to determine whether hyperactive KRAS signaling in LECs can cause mice to develop a phenotype that resembles GSD. We bred *Prox1-CreER^T2^* mice with *Kras^wt/LSL-G12D^* mice to generate control (*iLEC^Ctrl^*) and *Prox1-CreER^T2^*;*Kras^wt/LSL-G12D^* (*iLEC^Kras^*) mice (Figure 3). *Prox1-CreER^T2^* mice express a tamoxifen-inducible Cre in LECs and *Kras^LSL-G12D^* mice express an active form of KRAS (p.G12D) in Cre-positive cells. We decided to use the *Kras^wt/LSL-G12D^* strain, because it has been widely used by others to generate genetically engineered mouse models of diseases caused by hyperactive KRAS signaling, such as lung adenocarcinoma and pancreatic ductal adenocarcinoma (13, 14). The G12D mutation is an oncogenic mutation, like the G12V mutation, and is present in COSMIC 15,848 times. Femurs and tibias were collected from tamoxifen-treated mice and immunostained for Lyve1 and podoplanin (Figure 3). We found that bones from *iLEC^Ctrl^* mice did not contain lymphatics (*n* = 10 mice; Figure 3). In contrast, bones from 2 of 3 *iLEC^Kras^* mice contained lymphatics (Figure 3). This finding shows that *iLEC^Kras^* mice exhibit a phenotype similar to patients with GSD.

### iLEC^Kras^ mice have fewer lymphatic valves than iLEC^Ctrl^ mice.

**Figure 4:**
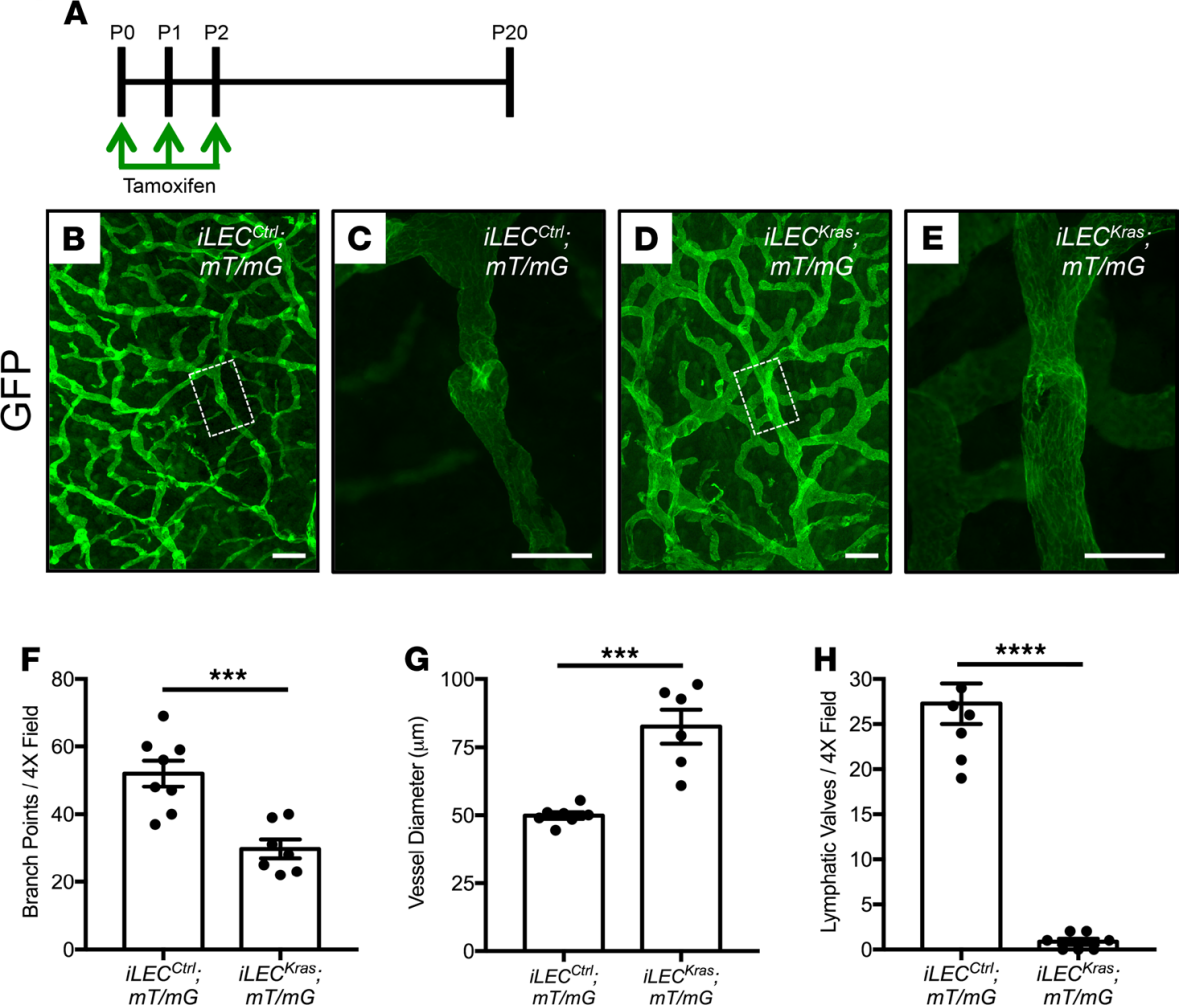
*iLEC^Kras^* mice have fewer lymphatic valves compared with *iLEC^Ctrl^* mice. (**A**) Schematic showing when mice were fed tamoxifen (2 μl of 25 mg/ml solution). Tissues were collected on P20. (**B–E**) Representative images of GFP-stained ear skin whole mounts from *iLEC^Ctrl^*;*mT/mG* mice (**B** and **C**) and *iLEC^Kras^*;*mT/mG* mice (**D** and **E**). (**C**) A higher-magnification view of the boxed region in **B**, showing a lymphatic valve with a normal V-shaped morphology in an *iLEC^Ctrl^*;*mT/mG* mouse. (**E**) A higher-magnification view of the boxed region in **D**, showing a lymphatic in an *iLEC^Kras^*;*mT/mG* mouse. This lymphatic does not have a valve with a normal V-shaped morphology. (**F**) *iLEC^Ctrl^*;*mT/mG* mice had significantly more lymphatic branch points per 4× field (52 ± 3.845; *n* = 8) than *iLEC^Kras^*;*mT/mG* mice (29.71 ± 2.775; *n* = 7). (**G**) *iLEC^Ctrl^*;*mT/mG* mice had significantly skinnier lymphatics (49.91 ± 1.232 μm; *n* = 7) than *iLEC^Kras^*;*mT/mG* mice (82.59 ± 6.178 μm; *n* = 6). (**H**) *iLEC^Ctrl^;mT/mG* mice had significantly more lymphatic valves per 4× field (27.25 ± 2.25; *n* = 8) than *iLEC^Kras^*;*mT/mG* mice (0.8571 ± 0.3401; *n* = 7). Data are presented as mean ± SEM. ***P < 0.001, ****P < 0.0001; unpaired Student’s *t* tests. Scale bar: 200 μm (**B** and **D**); 100 μm (**C** and **E**).

During our experiments we found that *iLEC^Ctrl^* mice lived significantly longer than *iLEC^Kras^* mice and that the median survival of *iLEC^Kras^* mice was only 26 days (Supplemental Figure 1; supplemental material available online with this article; https://doi.org/10.1172/jci.insight.149831DS1). On necropsy, we found that all *iLEC^Kras^* mice had chylothorax. Complications caused by chylothorax are the leading cause of death of patients with GSD (4). Because chylothorax can be caused by lymphatic valve defects, we analyzed lymphatic valves in *iLEC^Ctrl^* and *iLEC^Kras^* mice. In this experiment we included the *mT/mG* reporter strain in our breeding scheme to mark LECs that had undergone Cre-mediated recombination (Figure 4). The *mT/mG* reporter causes Cre-positive cells and their descendants to express GFP, while nonrecombined cells express tdTomato. Newborn mice were fed tamoxifen from P0 to P2 and ear skin was collected on P20 for whole-mount immunofluorescence staining for GFP (Figure 4). We found that the lymphatic network in *iLEC^Kras^*;*mT/mG* mice had significantly fewer branch points than the network in *iLEC^Ctrl^*;*mT/mG* mice (Figure 4). Additionally, the diameter of lymphatics was significantly greater in *iLEC^Kras^*;*mT/mG* mice than in *iLEC^Ctrl^*;*mT/mG* mice (Figure 4). Importantly, *iLEC^Kras^*;*mT/mG* mice had significantly fewer lymphatic valves than *iLEC^Ctrl^*;*mT/mG* mice (Figure 4).

**Figure 5:**
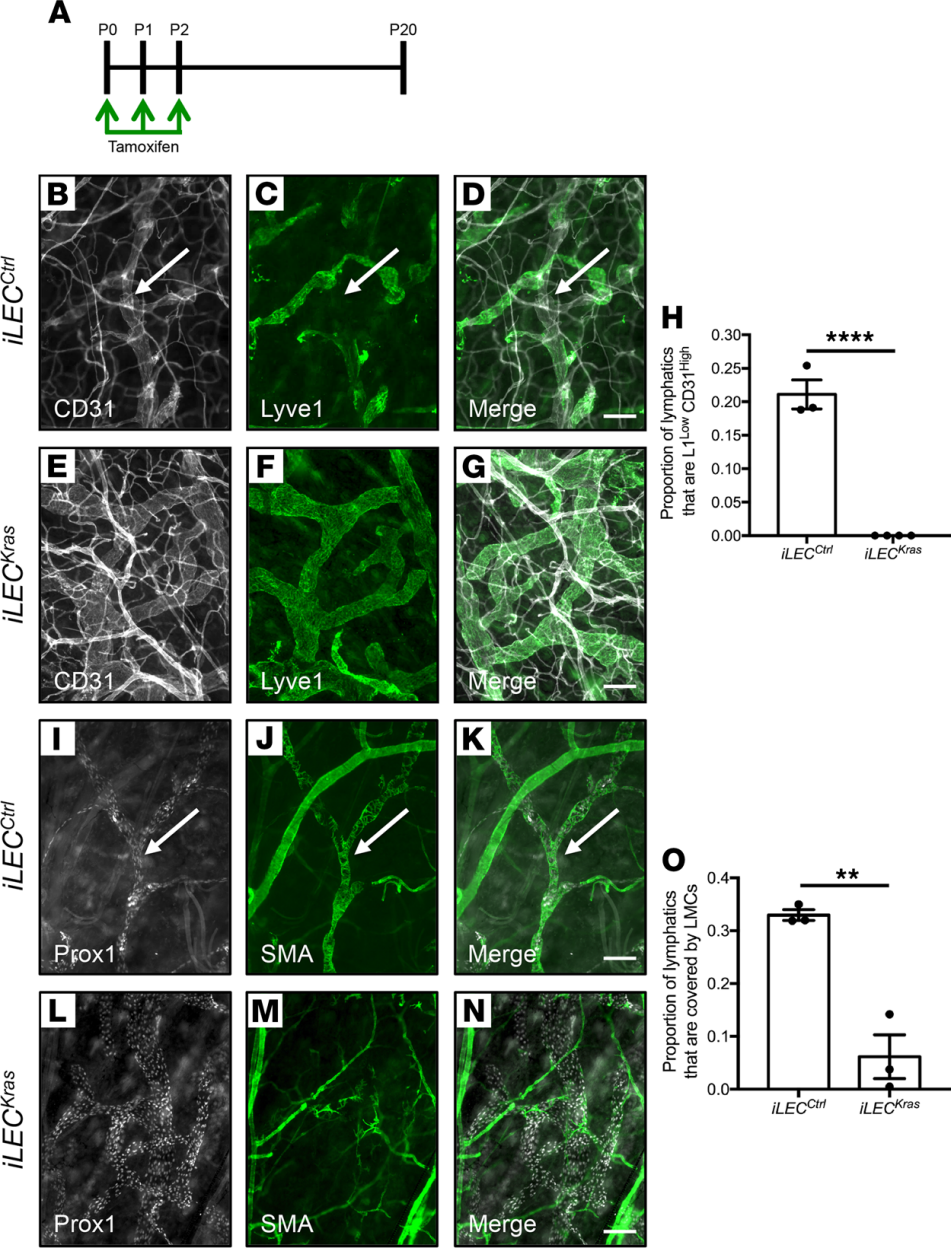
*iLEC^Kras^* mice have fewer lymphatics exhibiting characteristics of collecting vessels compared with *iLEC^Ctrl^* mice. (**A**) Schematic showing when mice were fed tamoxifen (2 μl of 25 mg/ml solution). Tissues were collected on P20. (**B–G**) Representative images of ear skin whole mounts from *iLEC^Ctrl^* mice (**B–D**) and *iLEC^Kras^* mice (**E–G**) stained with antibodies against CD31 and Lyve1. The arrows point to a Lyve1^lo^CD31^hi^ lymphatic in an *iLEC^Ctrl^* mouse. (**H**) The proportion of lymphatics that were Lyve1^lo^CD31^hi^ (see Methods for formula) was significantly greater in *iLEC^Ctrl^* mice (0.211 ± 0.02152; *n* = 3) compared with that in *iLEC^Kras^* mice (0 ± 0; *n* = 4). (**I–N**) Representative images of ear skin whole mounts from *iLEC^Ctrl^* mice (**I–K**) and *iLEC^Kras^* mice (**L–N**) stained with antibodies against Prox1 and SMA. The arrows point to a Prox1-positive lymphatic covered by SMA-positive lymphatic muscle cells (LMCs) in an *iLEC^Ctrl^* mouse. (**O**) The proportion of Prox1-positive lymphatics that were covered by SMA-positive LMCs (see Methods for formula) was significantly greater in *iLEC^Ctrl^* mice (0.3297 ± 0.01017; *n* = 3) compared with that in *iLEC^Kras^* mice (0.06143 ± 0.04137; *n* = 3). Data are presented as mean ± SEM. **P < 0.01, ****P < 0.0001; unpaired Student’s *t* tests. Scale bar: 100 μm.

Lymphatic valves are bicuspid structures that are present in collecting lymphatics (15). Because lymphatics in *iLEC^Kras^* mice did not contain valves, we set out to determine whether lymphatics in *iLEC^Kras^* mice lacked other defining characteristics of collecting lymphatics. Collecting lymphatics are Lyve1^lo^CD31^hi^ vessels that are surrounded by lymphatic muscle cells (LMCs) (16). We found that *iLEC^Ctrl^* mice had significantly more Lyve1^lo^CD31^hi^ lymphatics than *iLEC^Kras^* mice (Figure 5). We also found that *iLEC^Ctrl^* mice had significantly more lymphatics covered by LMCs compared with *iLEC^Kras^* mice (Figure 5).

### Lymphatic valves regress in iLEC^Kras^ mice.

**Figure 6:**
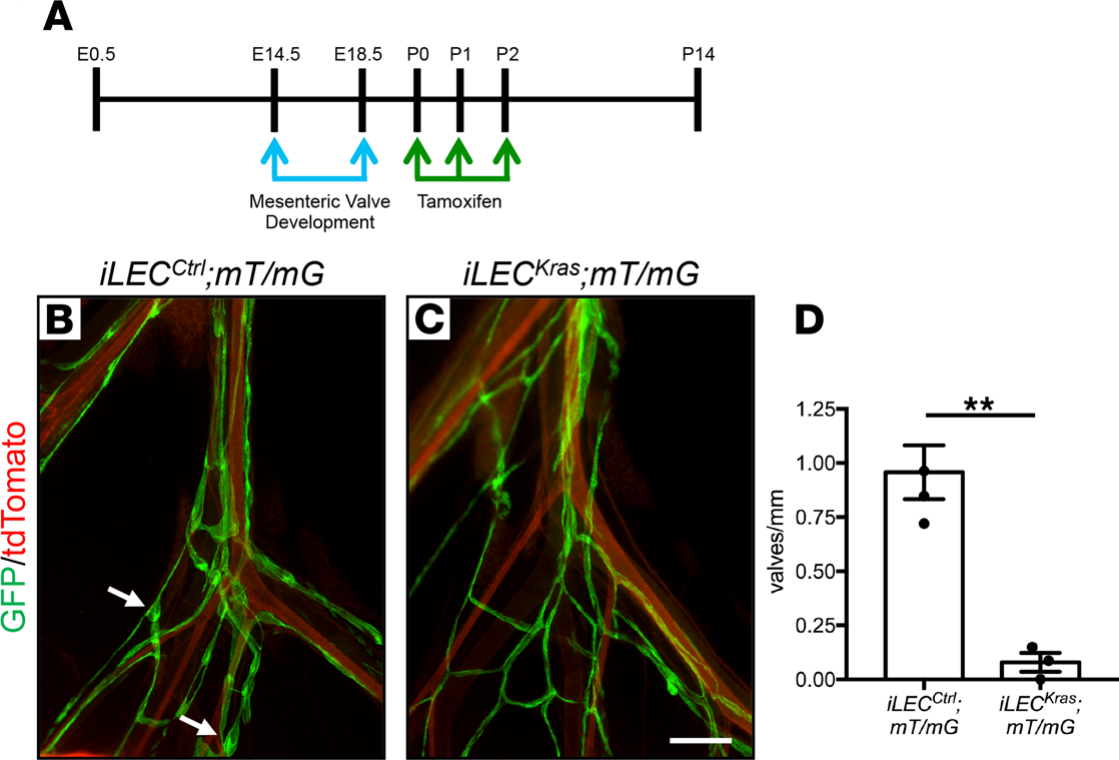
Existing lymphatic valves regress in *iLEC^Kras^* mice. (**A**) Schematic showing when mice were fed tamoxifen (2 μl of 25 mg/ml solution). Tissues were collected on P14. (**B** and **C**) Whole-mount preps of mesentery from *iLEC^Ctrl^*;*mT/mG* and *iLEC^Kras^*;*mT/mG* mice. The arrows point to examples of a lymphatic valve. (**D**) *iLEC^Ctrl^*;*mT/mG* mice had more lymphatic valves per mm lymphatic vessel length (0.9575 ± 0.1245; *n* = 4) than *iLEC^Kras^*;*mT/mG* mice (0.07877 ± 0.04322; *n* = 3). Data are presented as mean ± SEM. **P < 0.01; unpaired Student’s *t* tests. Scale bar: 300 μm.

Valves form in mesenteric lymphatics during embryonic development and exhibit a normal V-shaped morphology by E18.5 (17). To determine whether excessive KRAS signaling in LECs causes mesenteric lymphatic valve regression, we fed *iLEC^Ctrl^*;*mT/mG* and *iLEC^Kras^*;*mT/mG* mice tamoxifen from P0 to P2 and analyzed mesenteries on P14 by whole-mount immunofluorescence staining for GFP (Figure 6). We found that *iLEC^Ctrl^*;*mT/mG* mice had significantly more lymphatic valves than *iLEC^Kras^*;*mT/mG* mice (Figure 6). This suggests that excessive KRAS signaling in LECs causes lymphatic valve regression.

### Lymph moves in a retrograde manner in iLEC^Kras^ mice.

**Figure 7:**
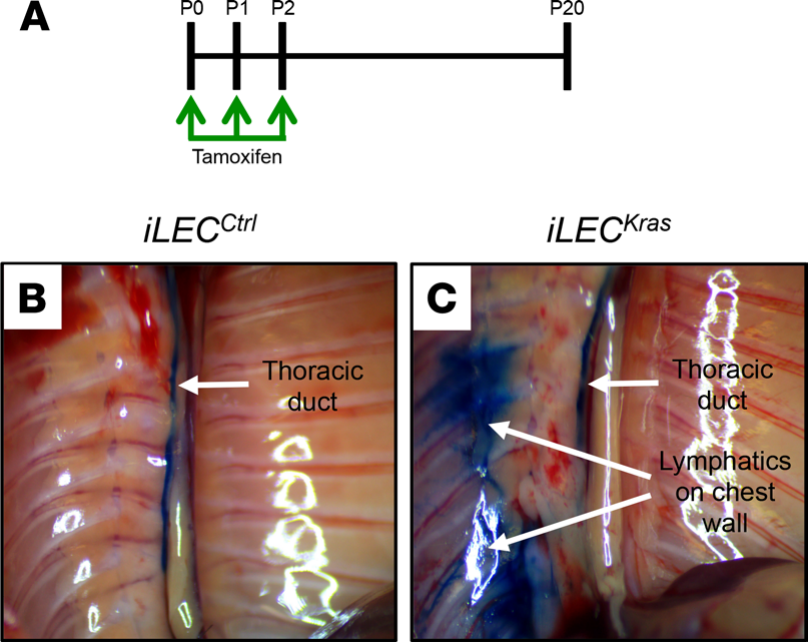
Lymphatics in *iLEC^Kras^* mice do not function properly. (**A**) Schematic showing when mice were fed tamoxifen (2 μl of 25 mg/ml solution). Lymphatics were imaged on P20. (**B**) The thoracic duct in *iLEC^Ctrl^* mice (*n* = 4) filled with Evans blue dye (arrow). (**C**) The thoracic duct and lymphatics on the chest wall filled with Evans blue dye (arrows) in 4 of 6 *iLEC^Kras^* mice.

Lymphatic valves prevent the retrograde flow of lymph (15). Retrograde lymph flow is reported to occur in GSD (18–21). To determine whether the loss of lymphatic valves in *iLEC^Kras^* mice affected the forward flow of lymph, we assessed lymphatic function in mice by intranodal lymphangiography. Newborn mice were fed tamoxifen from P0 to P2, and intranodal lymphangiography was performed on P20 by injecting Evans blue dye directly into the mesenteric lymph node (Figure 7). We found that Evans blue dye was confined to the thoracic duct in *iLEC^Ctrl^* mice (Figure 7). In contrast, Evans blue dye refluxed from the thoracic duct into intercostal lymphatics in *iLEC^Kras^* mice (Figure 7). This result suggests that excessive KRAS signaling in LECs causes retrograde lymph flow.

### Trametinib prevents lymphatic valve regression in iLEC^Kras^ mice.

**Figure 8:**
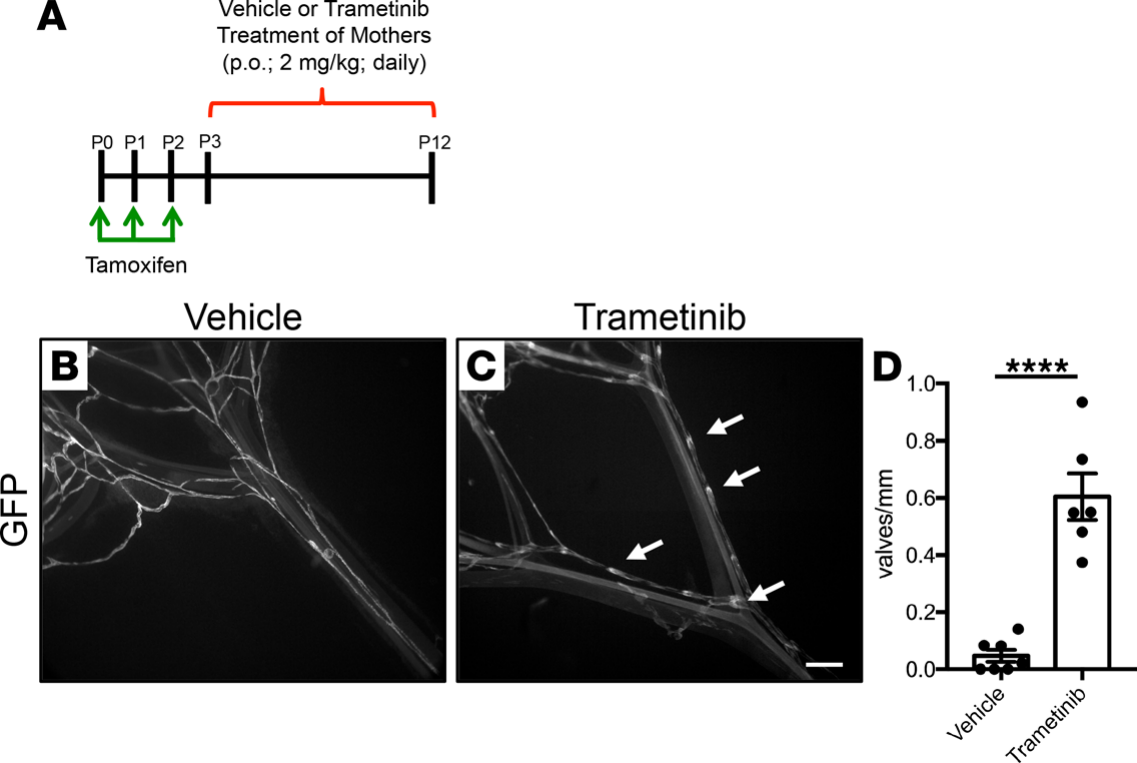
Trametinib prevents lymphatic valve regression in *iLEC^Kras^* mice. (**A**) Schematic showing when newborn mice were fed tamoxifen (2 μl of 25 mg/ml solution) and when their mothers received vehicle or trametinib (2 mg/kg; oral gavage; daily). This allowed pups to receive vehicle or trametinib by nursing. (**B** and **C**) Whole-mount preps of mesentery from vehicle or trametinib-treated *iLEC^Kras^*;*mT/mG* mice. The arrows point to examples of a lymphatic valve. (**D**) Vehicle-treated mice had significantly fewer lymphatic valves per mm of lymphatic vessel length (0.04686 ± 0.02098; *n* = 7) compared with trametinib-treated mice (0.6045 ± 0.08189; *n* = 6). Data are presented as mean ± SEM. ****P < 0.0001; unpaired Student’s *t* tests. Scale bar: 300 μm.

Trametinib is an FDA-approved MEK1/2 inhibitor that is used to treat cancers caused by hyperactive RAS/MAPK signaling, such as melanoma, non–small cell lung cancer, and anaplastic thyroid cancer (22–24). Because most *iLEC^Kras^* mice die before they have significant bone involvement, we analyzed the effect of trametinib on lymphatic valve regression in *iLEC^Kras^*;*mT/mG* mice. We fed newborn *iLEC^Kras^*;*mT/mG* mice tamoxifen from P0 to P2 and then used a transmammary route of administration to give pups vehicle or trametinib from P3 to P12 (Figure 8). This allowed pups to receive either treatment by nursing. We analyzed mesenteries on P12 by whole-mount immunofluorescence staining for GFP and found that trametinib-treated *iLEC^Kras^*;*mT/mG* mice had significantly more lymphatic valves than vehicle-treated *iLEC^Kras^*;*mT/mG* mice (Figure 8). This result demonstrates that trametinib can suppress lymphatic valve disintegration in *iLEC^Kras^*;*mT/mG* mice.

## Discussion

Despite recent advances in GSD research, the etiology of GSD has remained poorly understood. In the present study, we report the identification of a somatic activating mutation in *KRAS* (*c.35G>T*, p.G12V) in a tissue sample from a patient with GSD. The tissue was resected from a bone invested with abnormal lymphatics. We also developed and characterized a mouse model of GSD. We showed that *iLEC^Kras^* mice develop ectopic lymphatics in bone, lymphatic valve defects, and chylothorax. We also showed that trametinib can prevent lymphatic valve disintegration in *iLEC^Kras^* mice. These findings provide important clues to the etiology of GSD and suggest that MEK inhibitors could be effective at treating this disabling, disfiguring, and life-threatening disease.

Concurrent with our studies, another somatic activating mutation in *KRAS* (p.Q61R) was identified in a patient with GSD (25). The *KRAS* (p.Q61R) mutation is also a known hotspot mutation that causes hyperactive KRAS/MAPK signaling and is frequently observed in cancers (25). There is growing evidence that other complex lymphatic anomalies are associated with RAS pathway–activating mutations. Mutations in *RASA1* (26), *EPHB4* (27), *ARAF* (28), *NRAS* (29, 30), and *CBL* (31) have been identified in complex lymphatic anomaly patients. Additionally, patients with Noonan syndrome with RAS signaling pathway mutations can have lymphatic dysplasia, lymphedema, and retrograde lymph flow (32–34). Thus, GSD seems to be part of this wide spectrum of lymphatic dysplasias caused by abnormal RAS signaling. Although we report that our patient with GSD has a somatic activating mutation in *KRAS*, other genetic mutations could also cause this phenotypically heterogenous disease.

Bones in healthy individuals do not have lymphatics. However, patients with GSD develop ectopic lymphatics in bone (3). We previously showed that excessive PI3K signaling in LECs (*Prox1-CreER^T2^*;*LSL-Pik3ca^H1047R^* mice) or overexpression of VEGF-C by bone cells (*Osx-tTA;TetO-Vegfc* mice) stimulates the formation of lymphatics in bone (35, 36). Lineage-tracing studies with *Osx-tTA*;*TetO-Vegfc* and *Prox1-CreER^T2^*;*LSL-Pik3ca^H1047R^* mice revealed that LECs in bone arise from preexisting LECs located outside of bone (37). Additionally, we found that regional lymphatics grow, breach the periosteum, and then invade bone in *Osx-tTA*;*TetO-Vegfc* mice (37). Here, we report that excessive KRAS signaling in LECs also stimulates the formation of lymphatics in bone. Bone lymphatics in *iLEC^Kras^* mice could develop in a similar manner to bone lymphatics in *Osx-tTA*;*TetO-Vegfc* and *Prox1-CreER^T2^*;*LSL-Pik3ca^H1047R^* mice. Unfortunately, most *iLEC^Kras^* mice develop chylothorax and die before they have significant bone involvement, hindering more detailed study. Therefore, our future work will focus on optimizing the model so we can further study the mechanisms by which KRAS signaling promotes the development of lymphatics in bone.

Our understanding of the cellular and molecular mechanisms regulating lymphatic valve morphogenesis has increased in recent years (38). Lymphatic valve development begins when clusters of LECs upregulate the transcription factors Prox1, Foxc2, and Gata2 (38). These valve-forming cells elongate, reorient with respect to the longitudinal axis of the vessel, collectively invade the vessel, and then form a bicuspid valve comprised of 2 layers of LECs (38). Interestingly, the loss of *EphbB4* or *Rasa1* (encoding for p120RasGAP protein) increases RAS/MAPK signaling and impairs lymphatic valve development (39, 40). We found that hyperactive Kras signaling in LECs also impairs the development and maintenance of lymphatic valves. These findings provide further evidence that the RAS/MAPK signaling pathway serves a critical role in the development and maintenance of lymphatic valves, although the precise mechanisms remain unclear.

The identification of actionable mutations in complex lymphatic anomaly patients is ushering in a new era of precision medicine for the treatment of these life-threatening diseases. Trametinib is an FDA-approved MEK inhibitor that has been used to treat diseases caused by excessive RAS/MAPK signaling. To our knowledge, there are only 2 case reports of complex lymphatic anomaly patients with a RAS pathway–activating mutation being treated with trametinib (28, 31). Importantly, the health of these 2 patients improved in response to trametinib but not to sirolimus (28, 31). The precise mechanism by which trametinib improved lymphatic function in these patients was not determined. We found that trametinib prevents the loss of lymphatic valves in *iLEC^Kras^* mice. This finding reveals one mechanism by which trametinib could improve lymphatic function in patients with RAS pathway–activating mutations and supports the testing of MEK inhibitors in patients with GSD.

In conclusion, we have identified an activating somatic mutation in *KRAS* as a cause of GSD in a severely affected patient. We show that active KRAS signaling in LECs impairs development of lymphatic valves and stimulates development of lymphatics in bone. We also show that trametinib prevents valve loss caused by hyperactive KRAS signaling in LECs. These findings give a basis for testing MEK inhibitors as treatment in GSD and strengthen the idea for using RAS pathway inhibitors in other complex lymphatic anomalies caused by mutations in the RAS/MAPK pathway.

## Methods

### Sample collection.

Clinical data were collected via a standard questionnaire. The referring clinician evaluated the phenotype of this patient. DNA was extracted from blood and frozen tissue using a Wizard genomic DNA purification kit (Promega).

### Immunohistochemistry of human samples.

Endogenous peroxidase activity was blocked with 3% H_2_O_2_, and slides were stained with the following primary mouse monoclonal antiantibodies: CD31 (clone JC70A, Dako-Agilent; dilution 1:70), CD34 (clone QBEnd-10, Dako-Agilent; dilution 1:24), D2-40 (clone D2-40, Dako-Agilent; dilution 1:90), and SMA (clone BS66, Nordic Biosite; dilution 1:100). Antigen retrieval was performed using CC1 buffer (Ventana-Roche) for 36 minutes for the monoclonal antibodies and CC2 buffer (Ventana-Roche) for 68 minutes for the polyclonal antibody on ultraView automated systems instruments (Ventana-Roche).

### Targeted next-generation sequencing.

DNA was screened by Ion Torrent technology using a comprehensive cancer panel containing 408 cancer-related genes (Ion AmpliSeq Designer, Thermo Fisher Scientific). Libraries were prepared using the Ion AmpliSeq Library Kit, according to the manufacturer’s protocols (Life Technologies), starting with 10 ng DNA for each of the 2 primer pools. Sequencing was performed on an Ion Proton (Genomics platform of University of Louvain). Sequences were aligned to the human reference genome (hg19) by the Ion Torrent Suite Server 5 (Life Technologies) to generate.*bam* files. Variants were then called through the Torrent variant caller (version 5.8.0). Filtering of variants was performed using our in-house developed Highlander interface (http://sites.uclouvain.be/highlander/).

### WES and variant calling.

DNA samples also underwent WES with the SureSelect v6 capture kit and Illumina-based sequencing. We achieved a median vertical coverage of 73× for blood and 256× for tissue. Sequences were aligned to the reference human genome (hg19) and processed with Highlander, a software program developed in our laboratory. It has a user-friendly graphical interface and uses open source algorithms, such as Picard for removal of duplicates and recalibration of quality values, and GATK for variant calling. Frequencies in the population, presence in Cosmic, and impact prediction by Sift, SNPeff, Mutation Taster, PolyPhen2_hdiv, PolyPhen2_hvar, Lrt and Mutation Assessor and Deogen2 were used to filter variants of interest. We kept variants reported at a frequency of ≤0.001 in gnomAD (https://gnomad.broadinstitute.org) and variants that are predicted to affect splicing or predicted damaging by at least 3 of 8 of the software programs. Variants that met these criteria were subsequently visually verified using the Integrative Genome Viewer (http://software.broadinstitute.org/software/igv/). As we sequenced blood and tissue of the patient**,** we also used Mutect2 (10) to search for somatic mutations (this improved algorithm allows subtraction of germline [blood] variants from those in tissues) as well as FACETS (11) and ExomeDepth (12) to search for copy number alterations in the WES data.

### Mice and genotyping.

Mice were maintained in ventilated microisolator cages and were fed a standard diet ad libitum. Mice were provided igloos and nestlets as enrichment items. Both male and female mice were used in experiments. Littermates were used as controls for experiments. *Prox1-CreER^T2^* mice (41) were genotyped with the following primers: 5′-GTGGAAAGGAGCGTACACTGA-3′; 5′-CACACACACACACGCTTGC-3′; and 5′-GCCAGAGGCCACTTGTGTAG-3′. The wild-type allele was 370 bp and the Cre allele was 267 bp. The *mT/mG* mice (42) were genotyped with the following primers: 5′-CTCTGCTGCCTCCTGGCTTCT-3′; 5′-CGAGGCGGATCACAAGCAATA-3′; and 5′- TCAATGGGCGGGGGTCGTT-3′. The wild-type allele was 330 bp and the mutant allele was 250 bp. *Kras^LSL-G12D^* mice (13) were genotyped with the following primers: 5′-CTAGCCACCATGGCTTGAGT-3′ and 5′-TCCGAATTCAGTGACTACAGATG-3′. The mutant allele was 350 bp.

### Preparation of tamoxifen and trametinib.

Tamoxifen (25 mg; MilliporeSigma, T5648) was dissolved in a mixture of ethanol (100 μl; MilliporeSigma, E7023) and peanut oil (900 μl; MilliporeSigma, W530285). To induce Cre-mediated recombination, newborn mice were fed 2 μl of tamoxifen on P0, P1, and P2 with a P10 pipette. Trametinib (GSK1120212; 5 mg; Selleck, S2673) was dissolved in DMSO (1 ml; MilliporeSigma, D8779). We then added PEG300 (4 ml; MilliporeSigma, 8.07484), Tween 80 (500 μl; MilliporeSigma, P4780), and saline (4.5 ml; Baxter, 2F7124). Mice were administered vehicle or trametinib (2 mg/kg) with a 20-gauge gavage needle.

### Primary antibodies.

The following primary antibodies were used for immunohistochemistry or immunofluorescence staining: goat anti-Lyve1 (R&D Systems, AF2125; dilution 1:250 and 1:1000), chicken anti-GFP (Abcam, ab13970; dilution 1:1000), hamster anti-podoplanin (Abcam, ab11936; dilution 1:1000), rat anti-CD31 (BD Biosciences, 553370; dilution 1:1000), rabbit anti-Prox1 (Abcam, ab101851; dilution 1:500), and Cy3-conjugated mouse anti-SMA (MilliporeSigma, C6198; dilution 1:1000).

### Decalcification of bone.

Eight-week-old mice were euthanized and then perfused with PBS plus heparin and then with 4% paraformaldehyde (PFA). Femurs and tibias were fixed overnight in 4% PFA and then decalcified for 2 weeks in 10% EDTA (pH 7.4). Bones were then processed and embedded by the histology core at UT Southwestern Medical Center. Tissues were cut (5 μm thick) and placed on glass slides for immunohistochemistry.

### Immunohistochemistry and immunofluorescence staining of mouse tissue sections.

Slides were heated at 60°C for 30 minutes, deparaffinized with xylene, and then rehydrated through a descending ethanol series (100%–0%). A hydrogen peroxide/methanol solution was used to block endogenous peroxidase activity, and TBS plus 0.2% Tween 20 (TBST) plus 20% Aquablock was used to block nonspecific binding of antibodies. Slides were incubated overnight with primary antibodies diluted in TBST plus 5% BSA. Slides were washed with TBST and then incubated for 1 hour with secondary antibodies diluted in TBST plus 5% BSA. Antibody binding was detected with an ImmPACT DAB Peroxidase Substrate Kit (Vector, SK-4105). Slides were then dipped in hematoxylin and dehydrated, and coverslips were mounted with CytoSeal (Thermo Fisher Scientific, 8312-4).

### Whole-mount immunofluorescence staining.

Tissues were collected, fixed overnight in either 1% or 4% PFA, washed with PBS (6 times for 15 minutes each time), and then permeabilized with PBS plus 0.3% TX-100 (PBST). Nonspecific binding of antibodies was blocked with PBST plus 20% Aquablock (East Coast Bio, PP82-W0332). Tissues were incubated overnight with primary antibodies diluted in PBST. Tissues were washed (3 times for 40 minutes each time) with PBST and then incubated overnight with the appropriate secondary antibodies diluted in PBST. Tissues were washed again (3 times for 40 minutes each time) with PBST and then mounted on slides with ProLong Gold plus DAPI (Invitrogen, P36931).

### Quantification of lymphatic branch points.

Images of the medial portion of the ear were captured with a ×4 objective. The number of lymphatic branch points per microscopic field were then counted.

### Method to measure lymphatic vessel diameter.

A grid was placed over images captured with a ×4 objective. We then measured the diameter of lymphatics at points where the gridlines intersected on lymphatics. Between 11 and 42 measurements were taken for each ear. Values were averaged together for each ear to yield a final value for the ear.

### Quantification of lymphatic valves.

For ear skin, the medial portion of the ear was imaged with a 4× objective and then analyzed with NIS-Elements imaging software. The number of GFP^hi^ valves exhibiting a normal V-shape morphology were counted per microscopic field. For mesentery, loops of mesentery were mounted on slides, imaged with a ×4 objective, and the number of GFP^hi^ valves exhibiting a normal V-shape morphology were counted per mm of lymphatic vessel length.

### Method to determine the proportion of Lyve1^lo^CD31^hi^ lymphatics.

The medial portion of the ear was imaged with a 4× objective and then analyzed with NIS-Elements imaging software. A 35-line by 47-line multipurpose test grid, with a line length of 48 μm and a spacing of 48 μm (total number of intersecting points = 1645), was overlaid onto the images. The total test area was 3.77 mm^2^. We then counted the number of points that intersected on Lyve1^lo^CD31^hi^ lymphatics and the number of points that intersected on Lyve1^hi^CD31^hi^ lymphatics. These values were used to determine the proportion of lymphatics that were Lyve1^lo^CD31^hi^. The proportion of Lyve1^lo^CD31^hi^ lymphatics equaled the following: (the number of points that intersected on Lyve1^lo^CD31^hi^ lymphatics)/(the number of points that intersected on Lyve1^lo^CD31^hi^ lymphatics plus the number of points that intersected on Lyve1^hi^CD31^hi^ lymphatics).

### Method to determine the proportion of lymphatics covered by LMCs.

The medial portion of the ear was imaged with a ×4 objective and then analyzed with NIS-Elements imaging software. A multipurpose test grid with the same parameters described above was overlaid onto the images, and we counted the number of points that intersected on lymphatics covered by LMCs and the number of points that intersected on lymphatics not covered by LMCs. These values were used to determine the proportion of lymphatics that were covered by LMCs. The proportion of lymphatics covered by LMCs equaled the following: (the number of points that intersected on lymphatics covered by LMCs)/(the number of points that intersected on lymphatics covered by LMCs plus the number of points that intersected on lymphatics not covered by LMCs).

### Intranodal lymphangiography.

A 1% working solution of Evans blue dye was created by dissolving Evans blue dye (MilliporeSigma, E2129) in sterile PBS. Mice were anesthetized with an intraperitoneal injection of avertin and kept warm with a heating pad. A midline incision was made to expose the intestines, and Evans blue dye was injected directly into the mesenteric lymph node. The chest cavity was then opened and imaged.

### Statistics.

Data were analyzed using GraphPad Prism statistical analysis software (version 7.0). All results are expressed as mean ± SEM. The number of mice in each group is indicated in the figure legends (*n* = number of mice). For experiments with 2 groups, unpaired 2-tailed Student’s *t* tests were performed to test means for significance. A log-rank (Mantel-Cox) test was performed to test survival curves for significance and a Fisher’s exact test was performed to test the bone findings for significance. Data were considered significant at *P* < 0.05.

### Study approval.

The genetic studies were approved by the institutional review board of the University of Louvain (no. B403201629786), and the patient signed a written informed consent for participation and for publishing photographs. The animal experiments described in this manuscript were carried out in accordance with an animal protocol approved by the Institutional Animal Care and Use Committee of UT Southwestern Medical Center (APN, no. 2016-101510).

## Author contributions

lMB collected the patient samples and clinical data. CG performed the anatomopathological characterizations. PB and NHS performed the genetic analyses with the help of RH for Highlander, all under the supervision of MV. MV also provided funding for the studies on human samples. ALM and MTD designed and conducted the animal experiments and analyzed and interpreted results. MTD provided funding for the animal experiments. NHS, ALM, RH, CG, LMB, PB, MV, and MTD contributed to the writing of the manuscript. All authors read and approved the final manuscript.

## Supplementary Material

Supplemental data

## Version 1 06/22/2021

In-Press Preview

## Version 2 08/09/2021

Electronic publication
