# Supplementary material for: KRAS-driven model of Gorham-Stout disease effectively treated with trametinib
Source: JCI Insight. 2021 Aug 9;6(15):e149831. doi: 10.1172/jci.insight.149831 (PMC8410066; doi:10.1172/jci.insight.149831)
Supplement: Supplemental data [file jciinsight-6-149831-s197.pdf]

Supplemental Figure 1. A. Image of chyle from an $iLEC^{Kras}$ mouse. **B.** Survival curves for $iLEC^{Ctrl}$ mice (n = 23) and $iLEC^{Kras}$ mice (n = 13). Mice were fed tamoxifen (2 µl of 25 mg/ml solution) from P0-P2. The median survival of $iLEC^{Kras}$ mice was 26 days. $P < 0.0001$; log-rank (Mantel-Cox) test.

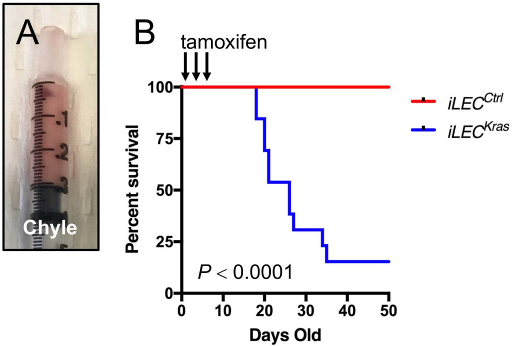
A
Chyle
B
tamoxifen
100
75
50
25
0
Percent survival
0
10
20
30
40
50
Days Old
$iLEC^{Ctrl}$
$iLEC^{Kras}$
$P < 0.0001$
